# Parental separation and behaviours that influence the health of infants aged 28 to 32 months: a cross-sectional study

**DOI:** 10.1186/s12887-018-1062-6

**Published:** 2018-02-27

**Authors:** Nadine Kacenelenbogen, Michèle Dramaix-Wilmet, M. Schetgen, M. Roland, Isabelle Godin

**Affiliations:** 1 Université Libre de Bruxelles, General Medicine Department, Campus Facultaire Erasme, Route de Lennik 808/612, 1070 Brussels, Belgium; 2 Université Libre de Bruxelles, Centre for Research in Epidemiology, Biostatistics and Clinical Research, School of Public Health, Campus Erasme CP598, Route de Lennik 808, 1070 Brussels, Belgium; 3 Université Libre de Bruxelles, Department of Health Information Promotion Education (SIPES), School of Public Health, Campus Erasme CP598, Route de Lennik 808, 1070 Brussels, Belgium

**Keywords:** Preschool children, Parental separation, Passive smoking, Prevention and screening

## Abstract

**Background:**

In Western countries, many children are affected by the separation of their parents.

The study’s main objective was to analyse the parental behaviours potentially influential for preschool children’s health by family structure (parents together or separated).

**Methods:**

We conducted a cross-sectional study based on data collected from examinations as part of free preventive medical consultations in the French Community of Belgium. During the assessment of 30,769 infants aged 28 to 32 months, information was collected on the parents’ use of tobacco, brushing of the infant’s teeth, being monitored by a dentist, and receiving vision screening. The chi^2^ test was applied and the odds ratios were derived to compare the two groups of children (exposed/not exposed to parental separation). Multivariate logistic regression analyses were used to adjust the effect of exposure.

**Results:**

Nearly one in ten (9.8%) did not live with both parents under the same roof. Taking into account the social and cultural environment and other potential confounders at our disposal, we found that in the event of parental separation, behaviours differ in comparison with situations where parents live together; the adjusted odds ratios (ORs) (95% confidence interval) for the infant’s exposure to tobacco, absence of teeth brushing, lack of monitoring by a dentist and absence of visual screening, were respectively 1.7 (1.2–2.0), 1.1 (0.9–1.2), 1.3 (1.1–1.6), 1.2 (1.1–1.2), and 1.2 (1.1–1.4).

**Conclusions:**

This study confirms the suspicion that parental separation is an independent risk factor for parental behaviours that negatively influence the infant’s health. If these results are confirmed, this it could affect the work of the family doctors and paediatricians, especially in terms of family support and information to parents.

## Background

In Belgium in 2011, the crude divorce rate was 2.9 per 1000 inhabitants, which is in line with in the rest of Europe, despite some North/South disparities. This rate is similar in a number of other countries in Europe (e.g. Denmark and Germany) [1] and in other continents (United States, Canada and Australia) [2–4]. Again in Belgium, in 2013, almost 80,000 people registered for legal cohabitation, the Belgian equivalent of registered or civil partnership, compared with 36,000 who declared the dissolution of their legal cohabitation (or 450 per 1000) [5]. Therefore, in Western countries, parental separation affects many minors. In Canada in 2011, 20% of people aged under 15 years were living with a single parent [6]. In the United Kingdom (UK) in 2001, 20% of people aged under 18 years did not live with both of their parents living as a couple [7]. According to a longitudinal study of 3000 households, 20% of children aged 0 to 16 years were living in a single-parent family or stepfamily in Belgium in 2002 [8].

In Flanders, a cross-sectional study carried out in 2013 showed that 10% of children under 2 years of age had experienced parental separation: this figure rises to 26% for all children aged 0 to 17 [9]. It is indeed expected that the older children become, the more likely they are to experience the separation of their parents. In Belgium in 2009, in the French-speaking community, 6.4% of children aged 7 to 11 months monitored by the *Office de la naissance et de l’enfance (ONE – Office for Births and Childhood – see ‘Methods’ section)* [10], did not live with two biological parents who were together as a couple, with the figure rising to 9% in children aged 28 to 32 months [11]. Lastly, a 2010 survey of more than 10,000 young Belgian French-speakers showed that more than 23% of children aged 10 to 12 years lived in either a stepfamily (10%) or a single-parent family (13%). That figure rises to 34% when children were aged between 13 and 19 years [12].^,^ [13]

Thus, using cumulative frequency, we estimate that parental separation affects more than 25% of young people aged under 18 years in Belgium. If we take into account the annual number of births for the last 17 years [14], at least 500,000 minors (of a total population of 11 million inhabitants) experience an officially recorded parental separation. Some authors describe a link between a child not living with parents who are together as a couple and a greater frequency of somatic, psychological, behavioural and academic problems. For instance, a cross-sectional American study that surveyed 102,000 families between 2002 and 2003 observed that, after adjusting for socio-economic levels, children presented significantly more oral, respiratory, trauma-related, behavioural and academic problems, as well as using specialised care more often, in the event of parental break-up [15]. In Spain, recent studies have indicated that for children and adolescents, parental separation represents a risk factor for their physical health, particularly genito-urinary, gastrointestinal, dermatological and neurological, in addition to exposure to violence and emotional or psychopathological disorders [16].^,^ [17] A national survey conducted in Belgium between 1992 and 2002 of 27,500 families confirmed this increase in risk factors when parents separated, such as the loss of contact with one of the parents, parental psychopathology, passive smoking or a materially less-advantaged environment. In that survey, the children of separated couples were more often absent from school or had fallen behind [8]. In 2006, a focus group-based qualitative study described the issues that hindered the work of Belgian general practitioners (GPs) while monitoring the children of separated parents. These included difficulties communicating with parents about the children’s health and barriers to monitoring the children medically, particularly for chronic diseases, or adherence to the immunisation schedule. Those GPs also described somatic and psycho-behavioural repercussions in the children following separation [18].

To our knowledge, little research has been specifically carried out on the association between separation or divorce and parental behaviour that may influence child health. We hypothesise that the behaviour of parents with regard to their child’s health may be different when they are separated compared with when they are together. A cross-sectional study of nearly 80,000 Belgian infants aged 7 to 11 months has already shown a significant association between a child not living with both parents and passive smoking, absence of breastfeeding and non-adherence to the immunisation schedule [19].

### Objectives

Our study’s primary objective was to assess parental behaviour regarding children’s health according to family structure (parents separated or together) in a cohort of preschool children (28 to 32 months). The secondary objective was to identify other factors of use to primary care medical practitioners that were associated with parental behaviours detrimental to child development. Our study is of use to primary care practice, as it makes it possible to better tailor informative and preventive action in families.

## Methods

### Study population

In the French-speaking community of Belgium, the ONE [20] offers free preventive monitoring of pregnant women and children up to the age of 6 years. Data collected during assessments is centralised in a computerised database. For children aged under 3 years, data is collected at birth in the maternity hospital, on arrival home, and, for those who are seen at the ONE, between 7 and 11 months, 16 and 20 months, and 28 and 32 months. For each encounter, social and demographic data, along with parenting behaviours, are recorded by a nurse, midwife or social worker. Specially trained paediatricians or GPs assess the child’s health status (including psychomotor development). Once filled in, the anonymised sheets are entered into the central database. This system makes it possible to evaluate and adapt medico-social policy during the perinatal period and early childhood. We studied the data from 30,769 children recorded in the ONE database between 2006 and 2012 for whom there existed a preventive health assessment at 28 to 32 months after birth.

### Assessment of main exposure

Family structure was divided into six categories: the two parents together, parents separated, child only sees one parent, the child is in a children’s home/ foster home, other situations (with grandparents or other parents) and unknown. A summary of the study sample, comprising 30,769 children, is provided in Table 5 (Appendix). For subsequent analyses, only parents who were together or separated (*n* = 28,871) were retained, with children who see only one parent falling under the second category; the parents of 2835 children (9.8%) were separated and those of 26,036 children (90.2%) lived together. Table 1 compares the socio-demographic characteristics of the two types of family structure.

**Table 1:** Sample description

Initial variable	%	New categories (*without *unknowns*)	%
Gender *n* = 30,769			
Boy	51.3	–	–
Girl	48.7		
Child’s age *n* = 30,707		–	
≤27 mo	7.3		
28–32 mo	88.9		
≥33 mo	3.8		
Birthweight *n* = 30,757		*n* = 30,757	
< 1000 g	0.1		
1000–1499 g	0.6		
1500–1999 g	1.2		
2000–2499 g	5.1	< 2500 g	7.0
2500–2999 g	19.6	≥2500 g	
3000–3499 g	39.9		93.0
3500–3999 g	25.0		
4000–4499 g	5.5		
≥4500 g	3.0		
BMI (kg/m^2^) at examination	percentiles	*n* = 29,120	
*n* = 29,120			
< 13.1	1.0		
< 13.9	5.0		
< 14.4	10.0		
< 15.2	25.0	p3-p97	91.8
< 16.0	50.0	>p97	6.3
< 17.0	75.0	<p3	1.9
< 18.0	90.0		
< 18.7	95.0		
< 20.9	99.0		
Mother’s age in years at childbirth *n* = 29,883		n = 29,883	
9–15	0.2		
16–17	0.7	< 18	0.9
18–24	18.3	18/30	59.0
25–30	40.8	31/37	33.0
31–37	32.9	≥38	7.1
38–44	6.9		
45–53	0.2		
Mother’s level of education *n* = 30,769		*n* = 24,530	
Did not complete primary school/no schooling	2.3		22.4
Completed primary school/did not complete lower secondary	4.3	< Upper secondary school	30.8
Completed lower secondary school	11.2	Completed upper secondary school	46.8
Completed upper secondary school	24.6	Completed third-level/university or not	
Completed third-level/university or not	37.3		
Unknown	20.3		
Mother’s occupation *n* = 15,038 (2006–9)		*n* = 7293	
Unemployed/stay-at-home	18.5	Unemployed/stay-at-home	
Part time	9.9		
Early retirement/invalidity/work incapacity	1.0	Early retirement/work incapacity/invalidity	38.1
On full-time career break/parental leave or similar	1.2	Student	2.1
Student	0.1	Works full time or part time/career break/parental leave	0.3
Works full time	17.7		59.5
Unknown	51.6		
Family income *n* = 15,038 (2006–9)		*n* = 7285	
No stable income	0.6		
1 or 2 incomes from benefits	5.5	No stable income	1.2
1 income from employment	16.3	1or 2 incomes from benefits	11.4
1 income from employment + 1	0.1	1 income from employment	33.6
income from benefits	0.3	2 incomes, of which ≥1 income from employment	53.8
Other (specify)	26.0		
2 incomes from employment	51.2		
Unknown			
Mother’s standard of French *n* = 16,990 (2006–9)			
None	1.8		
A little	8.1		
Very good	86.9		
Unknown	3.2	First language *n* = 28,582 (2006–12)	
Language spoken at home *n* = 13,783 (2010–12)			
Language other than French	34.3	French	77.6
French	53.8	Other	22.4
Unknown	11.9		
Goes to a nursery *n* = 13,783 (2010–12)		*n* = 12,040	
Yes	48.8		
No	38.5	Yes	55.9
Unknown	12.7	No	44.1
Family environment *n* = 30,769		*n* = 28,871	
Parents separated	7.5		
Only sees one parent	1.3		
Stepfamily	0.4	Parents separated/only sees one parent/stepfamily	9.8
Children’s home/home/foster home	0.6	Parents together	90.2
Grandparents, uncles/aunts, others	0.6		
Parents together	84.6		
Unknown	5.0		

### Assessment of other covariates

Using a ‘ready-made’ database (issued by the ONE), we selected the dependent variables that shed light on parental behaviour that is likely to influence the health of young children. However, the choice was limited as we could select only the variables that were available in this database. Therefore, the other independent variables retained for analysis were the mother’s age at childbirth, her level of education, her occupation and family income. The mother’s occupation and family income were mainly analysed to describe our sample, but they were not retained for creating the regression models because, as categories, they were ill suited to our research question. We placed stay-at-home mothers and those on benefits into as single occupational category, and those on early retirement or who had disabilities in another. Family income did not describe the level of earnings in euros but the type of income: for instance, families with one or two incomes from employment were in the same group, which partly covered the ‘couple’ variable (one or two parents) (Table 5 – Appendix). Our univariate analyses showed that the mother’s level of education was a good indicator of socio-economic status. This means that the higher the mother’s level of education, the more often they worked and had income from employment. Regarding language, two variables were available: the mother’s level of French (very good, a little, none) and the language spoken in the family (French, other language). Based on these two variables, we created the ‘first language’ variable (French, other language). We broke down the mother’s age, separating very young mothers (≤ 17 years) and older mothers (≥ 38 years) in particular. In addition, the child’s gender, birth weight and body mass index (BMI) were also analysed. ‘Unknown’ responses were eliminated for each of the variables taken into account: however, before doing this, we noted that the distributions of the variables relating to socio-economic status did not significantly differ among these ‘unknowns’. For multivariate analysis, the independent variable categories were grouped together according to the categories presented in the tables (Table 5 – Appendix).

### Outcome ascertainment

Dependent variables that were available and bore a relation to our research question were children’s being exposed to smoking on a daily basis between 2006 and 2009, brushing their teeth daily between 2006 and 2012, regularly seeing a dentist, and undergoing vision screening between 2010 and 2012. It ought to be noted that this vision-screening test has been made available since 2003 for all children monitored by the ONE at the 28–32-month examination. The aim is to detect functional amblyopia, which is the most common cause of unilateral visual impairment in children in Europe and the United States [21].

When treated between the ages of 2 and 3 years, amblyopia is curable, whereas it becomes permanent from the age of 6 years. Screening is performed using refractometry. It should be noted that, to take advantage of this free screening test by an orthoptist, parents must bring their child by appointment to a centre located at a different address to where the basic assessment takes place [22].

### Statistical analysis

The chi^2^ test was applied and the odds ratios (ORs) were derived to compare the two groups of children aged 28 to 32 months (exposed/not exposed to parental separation). Multivariate logistic regression analyses were used to adjust the effect of exposure. The models were designed using a backwards elimination method for potential confounders, and the variable of parental situation was automatically included in the models. Interactions between this variable and the other predictors were tested. The only interaction observed was for passive smoking, and it was between family structure and first language. The Hosmer–Lemeshow test was also used to check model fit. The absence of collinearity between the predictors included in the model was verified by means of variance inflation factors. The analyses were conducted using the STATA 12.0 software (http://www.stata.com).

## Results

In the 30,769 children, there were slightly more boys (51.3%) than girls (48.7%) and 7% of the children weighed less than 2500 g at birth (Table 5 – Appendix). In our sample, 1% of mothers were aged under 18 years at childbirth and 7% were aged 38 or over. Of the mothers, 22% had not completed secondary education. The percentage of mothers who did not have a job was 38%. French was not the first language in more than one in five families (Table 5 – Appendix). It should be noted that 9.8% of children did not live with both of their parents under the same roof (Table 5 – Appendix). The study of socio-demographic characteristics revealed significant differences between both types of families (Table 1). When parents did not live under the same roof, compared to non-separated parents, the mothers were more often younger than 18 years of age at childbirth (3.9% versus 0.5%) and less frequently had a higher level of education (28.9% versus 48.9%). We also noted that mothers who were separated from the child’s father were less likely to be French native speakers (18.1% versus 23.0%).

Between 2006 and 2009, 36% of children were exposed to smoking every day if the parents were separated compared with 20.7% when the parents were together (*p* < 0.001) (Table 2). Between 2006 and 2012, 9.3% of children did not have their teeth brushed daily when the parents were separated, compared with 7.7% when the parents lived together (*p* = 0.007). Regardless of family situation, between 2010 and 2012, more than 81% of the children had never had a check-up with a dentist (80.8% in couples living together), but the figure rose to 84.8% when the parents did not live together (*p* = 0.002). Lastly, again between 2010 and 2012, 48.2% of the children had not undergone vision screening when the parents were separated, compared with 40.7% when the parents were still together (*p* < 0.001) (Table 2). After adjusting for socio-economic and cultural factors, we observed that parental separation remained significantly associated with the variables having a potential impact on children’s health that were considered in this study, except for the absence of tooth brushing, the OR for which was no longer significant (OR 1.1–95% CI 0.9–1.2) (Table 3). Adjusted ORs were generally a little lower than the crude ORs, the highest observed being for passive smoking (OR 1.7–95% CI 1.5–2.0) (Table 3).

**Table 2:** Parental behaviour influencing the health of children aged 28 to 32 months

Variables of behaviour	Total	Parents together	Parentsseparated	P
≥ 1 person smokes in the home daily −2006-09 (n = 13,667)	(*n* = 2962)	(*n* = 2473)	(*n* = 489)	
% yes	22.2	20.7	36.1	
Crude OR (95% CI)		1	2.2 (1.9–2.4)	< 0.001
Daily tooth brushing −2006-12 (*n* = 25,976)	(*n* = 2046)	(*n* = 1851)	(*n* = 195)	0.007
% No	7.9	7.7	9.3	
Crude OR (95% CI)		1	1.2 (1.1–1.4)	
Sees the dentist −2010-12 (*n* = 10,686)	(*n* = 7826)	(*n* = 7054)	(*n* = 772)	0.002
% No	81.2	80.8	84.8	
Crude OR (95% CI)		1	1.3 (1.1–1.6)	
Vision screening −2010-12 (n = 12,725)	(*n* = 4923)	(n=	(*n* = 527)	< 0.001
% No	41.4	4396)	48.2	
Crude OR (95% CI)		40.7	1.4 (1.2–1.5)

**Table 3:** Behaviour of parents of children aged 28 to 32 months: adjusted ORs

	≥ 1 person smokes at home daily	Child does not brush his or her teeth daily	Child doesnot have a dentist	No vision screening
	(2006–09)	(2006–12)	(2010–12)	(2010–12)
Variable	OR (95% CI)	OR (95% CI)	OR (95% CI)	OR (95% CI)
Family structure				
Parents together	1	1	1	1
Parents separated	1.7 (1.5–2.0)	1.1 (0.9–1.2)	1.3 (1.1–1.6)	1.2 (1.1–1.4)
P	< 0.001	0.8	0.03	0.005
Mother’s level of education				
Third-level	1	1	1	1
Completed upper secondary	2.6 (2.3–2.9)	2.0 (1.7–2.3)	1.5 (1.3–1.7)	1.6 (1.4–1.8)
< Upper secondary	4.9 (4.3–5.6)	4.1 (3.6–4.7)	1.6 (1.4–1.9)	2.1 (1.8–2.4)
P	< 0.001	< 0.001	< 0.001	< 0.001
Mother’s age at childbirth				
< 18 years	1.7 (1.1–2.7)	0.9 (0.5–1.4)		
18/30 years	1	1		
31/37 years	0.9 (0.8–1.0)	1.1 (1.0–1.3)		
38 years and more	1.1 (0.9–1.3)	1.3 (1.1–1.6)		
P	0.03	0.02		
Mother speaks French				
Yes	1	1	1	1
No	0.7 (0.6–0.9)	1.5 (1.3–1.7)	1.2 (1.1–1.3)	1.5 (1.4–1.7)
P	< 0.001	< 0.001	0.02	< 0.001
≥ 1 parent allergic				
No	1			
Yes	1.2 (1.1–1.4)			
P	< 0.001			
Nursery attendance				
Yes			1	1
No			0.8 (0.7–0.9)	2.0 (1.8–2.3)
P			0.006	< 0.001
Birthweight				
≥2500 g	1		1	
< 2500 g	1.4 (1.2–1.8)		1.3 (1.1–1.7)	
P	< 0.001		0.02	
Age of the child				
≥33 mo		1	1	1
28–32 mo		0.9 (0.7–1.1)	1.4 (1.1–1.9)	1.3 (1.0–1.6)
≤ 27 mo		1.2 (0.9–1.6)	1.7 (1.2–2.5)	1.9 (1.4–2.6)
P		0.002	0.01	< 0.001
Grommets				
Yes				1
No				0.7 (0.5–0.9)
P				< 0.001
The child has eczema				
No	1			
Yes	0.9 (0.7–1.0)			
P	0.04			

The mother not having undergone higher education and young age (under 18 at childbirth) were significantly associated with exposure to smoking in children. However, when accounting for socio-economic status, passive smoking appeared to be rarer in families in which French was not the first language. A significant interaction was noted between family structure and first language: when French was the language spoken at home, parental separation became more strongly associated with exposure to smoking in children compare with parents living together (OR 1.9–95% CI 1.6–2.2), whereas family structure mattered little in non-French-speaking families (OR 0.7–95% CI 0.4–1.3) (Table 4). In addition, exposure to smoking was significantly associated with low birthweight (OR 1.4–95% CI 1.2–1.8). We also observed that the more parents suffered from allergic symptoms, the more smoking there was at home. Conversely, when a child suffered from eczema, there was less often exposure to smoking (Table 3).

**Table 4:** Interactions

Smoking at home*		
Family structure	First	First
	language: FR	language: ≠FR
	Adjusted OR	Adjusted OR
	(95% CI)	(95% CI)
Parents together	1	1
Parents separated	1.9 (1.6–2.2)	0.7 (0.4–1.3)
P	< 0.001	0.3

*Adjusted for mother’s level of education, child’s birthweight, child’s age on examination, and family’s allergy status

Regarding oral hygiene (tooth brushing and regular check-ups with a dentist), it seems that the more educated the mother was, and when French was the mother’s first language, the better oral hygiene was. We found a similar pattern with screening for amblyopia: the lower the mother’s level of education, and when French was not the first language, the less frequently children underwent screening (Table 3). Of course, the older a child was, the more they had had the opportunity to take advantage of dental and visual preventive check-ups. Also as expected, when a child attended a nursery accredited by the same organisation that manages vision screening (the ONE), they were more likely to undergo vision screening (Table 3).

## Discussion

Thus, adjusting for socio-economic and cultural factors as well as for the mother’s age at childbirth and certain characteristics of the children, we observed certain factors that were detrimental to children’s health more frequently when the parents did not live together under the one roof compared with when the parents were together.

### Passive smoking

Generally, smoking remains the primary risk factor of morbidity and mortality in Western countries; [23] in Belgium, we attribute 20,000 premature deaths (< 69 years) to smoking every year [24]. The health problems in children that are linked in the short term to smoking in their environment are well documented, including, besides the increased risk of sudden infant death syndrome [25], increased risk at all ages of upper [26] and lower [27] respiratory tract infections, as well as allergy symptoms [22]. In children aged 4 to 16 years, a correlation has also been described between cotinine levels and absenteeism from school, reduced respiratory function and wheezing [28]. In the longer term, nearly 17% of lung cancers in non-smokers may be attributable to high levels of passive smoking during childhood and adolescence [22, 29, 30]. Furthermore, in Belgium, parents who smoke double the rate of active smoking in their children [31]. Therefore, the main result of our study is that, regardless of the mother’s age or level of education, children were exposed to passive smoking significantly more often when their parents were separated than when their parents were together. From a public health perspective, we estimated at the community level the impact of family structure on smoking at home by assessing the population attributable fraction, which amounted to 14.9%. This is far from being negligible for a relatively commonplace situation. These observations are similar to those made in a cross-sectional study that analysed a large sample of Belgian infants aged 7 to 11 months: [19] between 2010 and 2012, exposure to smoking was more frequent in children when the parents were separated, with an OR of 1.5 (95% CI 1.3–1.7) adjusting for the mother’s age, occupation, and level of education. That confirms what has been said by Belgian GPs, who indeed offered an explanation for their observations: that separated parents exhibited more risky behaviours, including smoking, because of their anxiety [18]. Indeed, we find the link between separation, psychopathology,and parental smoking in other Belgian [8] and international [32, 33] studies. Consequently, our adjusted results that revealed more passive smoking in children when their parents did not live together appear to be consistent with those found in the literature. The association between passive smoking in children and parental separation seems particularly strong in families in which French was the first language, as we obtained an OR of approximately 2. In contrast, the risk was lower for children when French was not the language spoken at home, where the association between family structure and smoking disappeared (Tables 3 and 4). In the Wallonia-Brussels Federation, most non-Belgian families whose first language is not French are Muslim, and so come from a culture in which smoking habits are not necessarily comparable with those in the general population [34]. A recent systematic review has shown that smoking among immigrants coming from non-Western to Western countries was associated in men with a low level of education and with following their original way of life, but in women, conversely, the consumption of tobacco was associated with a high level of education and acculturation [35]. In our sample, 71% of women whose first language was not French had not finished or gone beyond secondary education (versus 18% of French-speaking mothers), which, according to the preceding study, is associated with a lower smoking rate in these foreign-born women.

### Oral hygiene

To a lesser extent than passive smoking, when parents were separated, we observed less optimal behaviour in children in terms of oral hygiene, namely less regular brushing of teeth (non-significant adjusted OR), but in particular less frequent visits to the dentist. We should point out that the ONE recommends a first visit to the dentist between the eruption of the first tooth and the age of 2 years at the latest, when most primary teeth are in place. This is consistent with the guidelines of the American Academy of Pediatrics, for example [36]. In our sample, there existed a positive association between the mother’s level of education and oral hygiene. The literature likewise associates social status and oral health [37, 38], but rarely family structure. In Brazil, not living with biological parents under the same roof may be more associated with poorer dental health, including cavities [39, 40]. The same observation has been made in the United States [15].

### Vision screening

We obtained similar results with vision screening as we did with oral hygiene. There exists a positive association between the mother’s level of education and children’s taking receiving this preventive service. That being said, children who did not live with parents who were still together received this screening less often. For vision screening and oral hygiene, it seems reasonable to interpret these results in light of the research (in Belgium, for example) that shows health differences according to economic, social and family status, particularly where access to preventive services is concerned [41].

### Strengths and weaknesses of the study

This study has a cross-sectional design, so the direction of the associations calculated between family structure and parental behaviour is unknown to us. Nevertheless, the fact that our analysis shows a statistical association between family structure and parental behaviour, and comparison with results provided by the literature, confirms for us that parents not living together constitutes at the very least an indicator of risk for the suboptimal behaviours studied here that influence children’s health. All socio-cultural strata were represented in our sample, thereby allowing us to build our regression models. However there was at least one selection bias. Families who bring their children to preventive check-ups at the ONE between 28 and 32 months are generally socially advantaged: 46% of the mothers had obtained a higher education qualification, whereas the figure does not exceed 30% in the general population [42] (Table 5 – Appendix). As national statistics in Belgium describe financial income in terms of euros per year, it is more difficult to compare the family incomes reported in our sample with those of the general population. However, the Brussels-Capital Health and Social Observatory [43], Wallonia Region [44], and the social integration services [45] report that benefits (unemployment or social protection benefits) are the only source of income for around 20% of the general population, which may be very cautiously compared with the 11% of families in our sample. This bias cannot be due to how the ONE operates, as it offers its services free of charge to all families, Belgian or foreign, regardless of immigration status. The positive association that persists in Belgium [41] between social status and use of preventive services is, in contrast, a possible explanation. This bias does not necessarily invalidate our results. The study population was relatively advantaged and, therefore, more likely to use preventive services, and so this confirms that the association between family structure (parents living together or not) and parental behaviour is independent of social status. We could even hypothesise that the selection bias minimised the ORs calculated. Lastly, our analysis arrived at expected conclusions, namely that the mother’s level of education and age at childbirth are predictors of behaviour in relation to health, which corroborates the other results found in our sample.

## Conclusion

### Implications for GPs

In French-speaking Belgium, in terms of prevention targeting children under three, our results reveal that, whatever the social environment, information work is necessary for all families regarding passive smoking, oral hygiene and vision screening*.* Our work confirms, however, that among the most precarious families, parental behaviour is less optimal. Here the role of the GP is essential; our statistics show that contacts with the family doctor increase with the level of poverty [46]. Moreover, our study shows that when parents are separated, parental behaviour is significantly poorer concerning the health of children aged under 3 years (passive smoking, oral hygiene and amblyopic screening) regardless of the level of maternal education or cultural environment. These results are echoed in other studies, which also show a greater risk of exposure to smoking, suboptimal nutrition and lower adherence to the immunisation schedule when infants did not live with two parents who were together [19]. In the event of parental separation, family doctors or paediatricians should be more focused on parental behaviour that may affect the health of their children such as interruption of contraception, pregnancy plans and/or postnatal follow-up. These are examples of situations where the GP could inform his patients at this level. The aim is not to stigmatize some families, but rather to better target health promotion work. Other studies, including prospective studies, should be conducted to better understand these public health challenges.

## Appendix

**Table 5:** Sociodemographic characteristics and family structure

Variable	Parents living together n (%)	Separated parents n (%)	P
Gender			0.240
Boy	13,271 (51.4)	1411 (50.2)	
Girl	12,549 (48.6)	1398 (49.8)	
Child’s age			0.006
≤27 mo	1805 (6.9)	238 (8.4)	
28–32 mo	23,223 (89.4)	2474 (87.5)	
≥33 mo	962 (3.7)	117 (4.1)	
Birth weight (g)			< 0.001
< 2500	1697 (6.5)	293 (10.3)	
≥2500	24,330 (93.5)	2542 (89.7)	
BMI – percentiles			0.797
p3-p97	22,687 (92.0)	2481 (92.0)	
>p97	1513 (6.1)	171 (6.3)	
<p3	457 (1.9)	46 (1.7)	
Mother’s age in years at childbirth			< 0.001
< 18	131 (0.5)	108 (3.9)	
18–30	14,964 (59.0)	1670 (60.7)	
31–37	8549 (33.7)	739 (26.8)	
≥38	1729 (6.8)	236 (8.6)	
Mother’s level of education			< 0.001
< Upper secondary school	4497 (20.5)	836 (36.3)	
Completed upper secondary school	6697 (30.6)	801 (34.8)	
Completed third-level/university or not	10,714 (48.9)	666 (28.9)	
Mother’s occupation (2006–9)			< 0.001
Unemployed/stay-at-home	2343 (36.6)	380 (50.7)	
Early retirement/work incapacity/invalidity	101 (1.6)	36 (4.8)	
Student	17 (0.3)	8 (1.1)	
Works full time or part time/career break/parental leave	3948 (61.6)	325 (43.4)	
Family income (2006–9)			< 0.001
No stable income	63 (1.0)	20 (2.6)	
1or 2 incomes from benefits	503 (7.9)	303 (40.0)	
1 income from employment	2000 (31.3)	395 (52.1)	
2 incomes, of which ≥1 income from employment	3834 (59.9)	40 (5.3)	
First language (2006–12)			< 0.001
French	19,412 (77.0)	2255 (81.9)	
Other	5813 (23.0)	499 (18.1)	
Goes to a nursery (2010–12)			0.927
Yes	5977 (55.8)	610 (56.0)	
No	4731 (44.2)	480 (44.0)	

## Abbreviations

BMI: Body mass index
ONE: Office of Birth and Childhood
OR: Odds ratio

## References

[1] Europa statistics explained. File: crude divorce rate, selected years, 1960–2011. http://ec.europa.eu/eurostat/statistics-explained/index.php?title=Special:ListFiles&user=Marcumc Accessed 17 Feb 2018.

[2] United States Census Bureau. The 2012 statistical abstract. Births, deaths, marriages, & divorces: marriages and divorces. https://www.census.gov/prod/2011pubs/12statab/vitstat.pdf. Accessed 17 Feb 2018.

[3] Statistics Canada. Description for Chart 8. Divorce and suicide rates, per 100,000, Canada, 1950 to 2008. http://www.statcan.gc.ca/pub/82-624-x/2012001/article/chart/11696-02-chart8-eng.htm. Accessed 17 Feb 2018.

[4] Australian Bureau of Statistics. Marriages and Divorces, Australia. 2013. http://www.abs.gov.au/ausstats/abs@.nsf/mf/3310.0. Accessed 17 Feb 2018.

[5] Statistics Belgium. Cohabitations légales.http://www.ibz.rrn.fgov.be/fr/registre-national/statistiques-de-population/. Accessed 17 Feb 2018.

[6] Statistique Canada. Portrait des familles et situation des cas particuliers dans les ménages au Canada. 2011. http://www12.statcan.gc.ca/census-recensement/2011/as-sa/98-312-x/98-312-x2011001-fra.cfm. Accessed 17 Feb 2018.

[7] Babb P, Bird C, Bradford B, Burtenshaw S, Gardener D, Howell S, et al. Social focus in brief: children 2002. National. Statistics. 2002; http://www.fairplayforchildren.org/pdf/1228009730.pdf. Accessed 17 Feb 2018.

[8] Petit S, Casman MT. Utilisation des données du PSBH pour mieux connaître les familles recomposées en Belgique: rapport de recherche. Fondation Baudouin. 2008. https://pure.fundp.ac.be/ws/files/12627193/pub2008_1839_l_enfantdanslafamillerecomposee.pdf. Accessed 17 Feb 2018.

[9] Vanassche S, Corijn M, Sodermans AK, Matthijs K (2013). Gezinstrajecten van ouders en kinderen na een (echt) scheiding.

[10] Office de la naissance et de l’enfance. http://www.one.be/presentation/about-us/. Accessed 17 Feb 2018.

[11] Fontaine L, Goetghebuer T, Liégeois M, Mauroy MC, Morales I, Rapport NES (2012). 2010; Banque de Données médico-sociales. Office de la naissance et de de l’enfance. Fédération Wallonie-Bruxelles.

[12] Decant P, de Smet P, Favresse D, Godin I. la sante des Élèves de 5e et 6e années primaires. 2013. Résultats de l’enquête HBSC 2010 en Fédération Wallonie-Bruxelles. https://dipot.ulb.ac.be/dspace/bitstream/2013/142427/1/HBSC_primaire_2013.pdf. Accessed 17 Feb 2018.

[13] Moreau N, Favresse D, de Smet P, Godin I. la sante des Élèves de l’enseignement secondaire. 2013. Résultats de l’enquête HBSC 2010 en Fédération Wallonie-Bruxelles. http://sipes.ulb.ac.be/index.php?option=com_mtree&task=att_download&link_id=165&cf_id=24. Accessed 17 Feb 2018.

[14] Statistics Belgium. Naissances et fécondité. Evolution du nombre de naissances en Belgique. 1830–2012. https://statbel.fgov.be/fr/themes/population/naissances-et-fecondite#figures. Accessed 17 Feb 2018.

[15] Bramlett MD, Blumberg SJ (2007). Family structure and Children’s physical and mental health. Health Aff.

[16] Seijo D, Fariña F, Corras T, Novo M, Arce R (2016). Estimating the epidemiology and quantifying the damages of parental separation in children and adolescents. Front Psychol.

[17] Martinón JM, Fariña F, Corras T, Seijo D, Souto A, Novo M (2017). Impacto de la ruptura de los progenitores en el estado de salud física de los hijos. European Journal of Education and Psychology.

[18] Kacenelenbogen N, Roland M, Schetgen M, Dusart AF (2013). The general practitioner and 510 children of separated parents in Belgium: a qualitative study and its implications. J Gen Pract.

[19] Kacenelenbogen N, Dramaix-Wilmet M, Schetgen M, Roland M (2014). Parental separation and behaviours that influence the health of infants aged 7-11 months: a cross-sectional study. BMJ Open.

[20] Office de la Naissance et de l’Enfance. Consultations pour enfants. http://www.one.be/index.php?id=2391. Accessed 17 Feb 2018.

[21] Levi DM, Knill DC, Bavelier D (2015). Stereopsis and amblyopia: a mini-review. Vis Res.

[22] Indicateurs de suivi des enfants à l’ONE. http://www.one.be/uploads/tx_ttproducts/datasheet/RA_BDMS_2002-2003_03.pdf. Accessed 17 Feb 2018.

[23] World Health Organization (2009). GLOBAL HEALTH RISKS GLOBAL HEALTH RISKS WHO mortality and burden of disease attributable to selected major risks.

[24] Peto R, Lopez A D, Boreham, Thun M. Mortality from smoking in developed countries 1950–2000. 2nd edition, revised June 2006. http://gas.ndph.ox.ac.uk/deathsfromsmoking/download%20files/Country%20presentations/Malta/Malta%20data.pdf. Accessed 17 Feb 2018.

[25] Carmona RH, Moritsugu KP, Williams RC, Near KA, Schoenfeld R, Gerberding JL, et al. The health consequences of involuntary exposure to tobacco smoke: a report of the surgeon general. US Department Of Health And human Services Public Health Service Office of the Surgeon General. 2006;709.

[26] Lieu JE, Feinstein AR (2002). Effect of gestational and passive smoke exposure on ear infections in children. Arch Pediatr Adolesc Med.

[27] Oberg M, Jaakkola MS, Woodward A (2011). Worldwide burden of disease from exposure tosecond-hand smoke: a retrospective analysis of data from 192 countries. Lancet.

[28] Mannino DM, Moorman JE, Kingsley B (2001). Health effects related to environmental tobacco smoke exposure in children in the United States: data from the third National Health and nutrition examination survey. Arch Pediatr Adolesc Med.

[29] Pang D, McNally R, Birch JM (2003). Parental smoking and childhood cancer: results from the United Kingdom childhood cancer study. Br J Cancer.

[30] Boffetta P, Trédaniel J, Greco A (2000). Risk of childhood cancer and adult lung cancer after 558 childhood exposure to passive smoke: a meta-analysis. Environ Health Perspect.

[31] Favresse D, De Smet P (2008). Tabac, alcool, drogues et multimédias chez les jeunes en Communauté française de Belgique. Résultats de l’enquête HBSC 2006. Service d’Information Promotion Education Santé (SIPES), ESP-ULB, Bruxelles.

[32] Clancy N, Zwar N, Depression RR (2013). Smoking and smoking cessation: a qualitative study. Fam Pract.

[33] Lawrence D, Hafekost J, Hull P, Mitrou F, Zubrick SR (2013). Smoking, mental illness and socioeconomic disadvantage: analysis of the Australian National Survey of mental health and wellbeing. BMC Public Health.

[34] Philippe H, Lucchini S. "Diversité sociolinguistique et ressources partagées. Regards critiques sur les politiques d’intégration linguistique en Belgique’." Revista de Sociolingüística. 2005. https://www.researchgate.net/profile/Silvia_Lucchini2/publication/28087857_Diversite_sociolinguistique_et_ressources_partagees_Regards_critiques_sur_les_politiques_d'integration_linguistique_en_Belgique/. Accessed 17 Feb 2018.

[35] Reiss K, Lehnhard J, Razum O (2015). Factors associated with smoking in immigrants from non579 western to western countries – what role does acculturation play? A systematic review. Tob Induc Dis.

[36] Hale KJ, American Academy of Pediatrics, Section on Pediatric (2003). Dentistry oral health risk assessment timing and establishment of the dental home. Pediatrics.

[37] Piovesan C, Antunes JL, Guedes RS, Ardenghi TM (2010). Impact of socioeconomic and clinical factors on child oral health-related quality of life (COHRQoL). Qual Life Res.

[38] Petersen PE (2005). Sociobehavioural risk factors in dental caries - international perspectives. Community Dent Oral Epidemiol.

[39] Paula JS, Leite ICG, Almeida AB, Ambrosano GMB (2012). 590 Pereira AC, Mialhe F L the influence of oral health conditions, socioeconomic status and home environment factors on schoolchildren's self-perception of quality of life. Health Qual Life Outcomes.

[40] Paula JS, Leite IC, de Almeida AB, Ambrosano GM, Mialhe FL (2013). The impact of socioenvironmental characteristics on domains of oral health-related quality of life in Brazilian schoolchildren. BMC Oral Health.

[41] Bossuyt N, Van Oyen H. Rapport de santé: Différences socio-économiques en santé. Institut Scientifique de la Santé Publique Service d’Epidémiologie IPH/EPI REPORTS N° 2001–013. https://www.wiv-isp.be/epidemio/epifr/santefr/sociofr.pdf. Accessed 17 Feb 2018.

[42] Stabel. La belgique en Chiffre. Census 2011 - Age, Situation sur le marché de l'emploi, Pays de citoyenneté, Niveau d'instruction, Lieu de résidence, Sexe, Sexe. https://statbel.fgov.be/fr/open-data/census-2011-age-situation-sur-le-marche-de-lemploi-pays-de-citoyennete-niveau-1. Accessed 17 Feb 2018.

[43] Observatoire de la santé et du Social Bruxelles. Baromètre social. Rapport bruxellois sur l’état de La pauvreté 2013 .http://www.ccc-ggc.irisnet.be/sites/default/files/documents/graphics/rapport-pauvrete/barometre_social_2013.pdf. Accessed 17 Feb 2018.

[44] Statistics Belgium. Taux d’emploi, taux de chômage, taux d’activité par sexe pour la Belgique et les régions, derniers 4 trimestres. https://statbel.fgov.be/fr/themes/emploi-formation/marche-du-travail/emploi-et-chomage. Accessed 17 Feb 2018.

[45] SPP Intégration Sociale. Bulletin statistique 2013.2. https://www.mi-is.be/sites/default/files/documents/bulletin_2013_septembre.pdf. Accessed 17 Feb 2018.

[46] Drieskens S, Gisle L. Enquête de Santé 2013 Rapport 3 : Utilisation des services de soins de santé et des services sociaux. Institut Scientifique de Santé Publique. Direction Opérationnelle Santé publique et surveillance. 2013. https://his.wiv-isp.be/fr/Documents%20partages/Summ_HC_FR_2013.pdf. Accessed 9 July 2017. Accessed 17 Feb 2018.

